# Characterisation of cylindrical curves

**DOI:** 10.1007/s00605-014-0705-4

**Published:** 2014-12-05

**Authors:** E. L. Starostin, G. H. M. van der Heijden

**Affiliations:** Department of Civil, Environmental and Geomatic Engineering, University College London, Gower Street, London, WC1E 6BT UK

**Keywords:** Curve on surface, Circular cylinder, Euclidean space, Moving frame, Curves of constant separation, Closed curves, 53A04, 53A05, 65D18

## Abstract

We employ moving frames along pairs of curves at constant separation to derive various conditions for a curve to belong to the surface of a circular cylinder.

## Introduction

While it is easy to find in the literature an ordinary differential equation characterising a spherical curve in terms of its curvature, torsion and their derivatives [2, 5], an analogous condition for a curve to belong to a cylinder of revolution seems to remain elusive in its explicit form, i.e., as a single equation involving only curvature, torsion and their derivatives. This is perhaps surprising given the simple nature of the surface. It was shown in [2] that a necessary condition may be formulated as a vanishing resultant of two auxiliary polynomials of the sixth and eighth degree with their coefficients depending on the curvature, torsion, their first derivatives and the second derivative of the curvature. Technically, the condition may be written out explicitly as a single polynomial of degree $\le$ $8^{8+6}$, but, clearly, doing this makes little sense. An earlier attempt to characterise cylindrical curves resulted in a system of two equations with an extra angular variable [3]. Here we present several alternative necessary and sufficient conditions for a curve to lie on a cylinder. We also allow for a slightly wider class of curves (that may contain straight pieces). A particularly elegant criterion is obtained in terms of the geometric properties of the curve as it lies in the surface, i.e., the geodesic torsion and geodesic and normal curvatures (Proposition 1). Some of the alternative criteria given here may be more convenient than existing criteria in certain applications (e.g, in computer vision). Our strategy is to regard a curve on a cylinder as a special case of two constant-separation curves, the second curve being the axis of the cylinder.

## Constant-separation curves and moving frames

Consider a regular $C^3$-curve ${{\mathbf {r}}}(s) \in {{\mathbb R}}^3, s\in [0,L]$, parametrised by arclength. We call this curve the *primary curve*. We denote by ${{\mathbf {t}}}(s)={{\mathbf {r}}}'(s)$ the unit tangent vector. Here and in the following a prime denotes differentiation with respect to $s$.

Let ${{\mathbf {d}}}(s)$ be a unit normal vector of differentiability class $C^2$. Given a constant $\rho \ne 0$ we define the *secondary curve* ${{\mathbf {a}}}(s) := {{\mathbf {r}}}(s)+\rho {{\mathbf {d}}}(s)$. The unit tangent to this curve is ${{\mathbf {t}}}_a(s)= \frac{{{\mathbf {a}}}'(s)}{|{{\mathbf {a}}}'(s)|}$. It is defined for $|{{\mathbf {a}}}'(s)|>0$. Clearly, ${{\mathbf {t}}}_a(s) \cdot {{\mathbf {d}}}(s) \equiv 0$. We say that ${{\mathbf {r}}}$ and ${{\mathbf {a}}}$ are constant-separation curves.

We define the following moving frames (all our frames are right-handed). First, for curves having nonvanishing curvature $\varkappa (s) := |{{\mathbf {t}}}'(s)| >0$, let $\{ {{\mathbf {t}}}(s), {{\mathbf {n}}}(s), {{\mathbf {b}}}(s) \}$ be the Frenet frame for the original curve, where ${{\mathbf {n}}}(s)={{\mathbf {t}}}'(s)/\varkappa (s)$ is the principal normal and ${{\mathbf {b}}}(s)={{\mathbf {t}}}(s) \times {{\mathbf {n}}}(s)$ the binormal. Next, we construct the unit vector ${{\mathbf {u}}}(s)={{\mathbf {t}}}(s) \times {{\mathbf {d}}}(s)$ to define the moving (or Darboux) frame $\{ {{\mathbf {t}}}(s), {{\mathbf {d}}}(s), {{\mathbf {u}}}(s) \}$. Finally, we define the analogous moving frame at the secondary curve $\{{{\mathbf {t}}}_a(s), {{\mathbf {d}}}(s), {{\mathbf {v}}}(s)\}$, where ${{\mathbf {v}}}(s) = {{\mathbf {t}}}_a(s) \times {{\mathbf {d}}}(s)$.

After choosing a coordinate system we may identify the orientations of the above three frames with elements of the group of orthogonal $3\times 3$ matrices:

$$\begin{aligned} R_{F}(s)&:= ({{\mathbf {t}}}(s), {{\mathbf {n}}}(s), {{\mathbf {b}}}(s) )\in SO(3) , \\ R(s)&:= ({{\mathbf {t}}}(s), {{\mathbf {d}}}(s), {{\mathbf {u}}}(s) )\in SO(3) , \\ R_{a}(s)&:= ({{\mathbf {t}}}_a(s), {{\mathbf {d}}}(s), {{\mathbf {v}}}(s) )\in SO(3) . \end{aligned}$$

These define three skew-symmetric $3\times 3$ matrices in the Lie algebra ${\mathfrak {so}}(3)$ as follows:1where we have introduced the ‘hat’ isomorphism between skew-symmetric matrices $${{\mathsf {\widehat{w}}} = \begin{pmatrix} 0 &{} -w_3 &{} w_2 \\ w_3 &{} 0 &{} -w_1 \\ -w_2 &{} w_1 &{} 0 \end{pmatrix}} $$ in ${\mathfrak {so}}(3)$ and rotation (or Darboux) vectors ${\mathsf {w}} = (w_1, w_2, w_3)$ in ${{\mathbb R}}^3$.

Thus we have defined three rotation vectors , where  is the rotation vector of the frame  is the rotation vector of the frame $\{ {{\mathbf {t}}}_{a}, {{\mathbf {d}}}, {{\mathbf {u}}} \}$ and  is the rotation vector of the Frenet frame $\{{{\mathbf {t}}}, {{\mathbf {n}}}, {{\mathbf {b}}} \}$ so that $\widetilde{\omega }_1 =\tau , \widetilde{\omega }_2 =0, \widetilde{\omega }_3=\varkappa$, where $\tau$ is the torsion of the primary curve ${{\mathbf {r}}}(s)$.

The orthonormal frames form a sequence under consecutive rotations about ${{\mathbf {t}}}$ and ${{\mathbf {d}}}$. Thus

2 $$\begin{aligned} R=R_{F} R_1(\xi ), \quad R_{a} = R R_2(\eta ), \end{aligned}$$

where

$$\begin{aligned} R_1(\xi )&= \exp (\xi \,{\mathsf {\widehat{e}}}_1) =\begin{pmatrix} 1 &{} 0 &{} 0 \\ 0 &{} \cos \xi &{} -\sin \xi \\ 0 &{} \sin \xi &{} \cos \xi \end{pmatrix} \in SO(3), \quad&{\mathsf {e}}_1&= (1,0,0), \\ R_2(\eta )&= \exp (\eta \,{\mathsf {\widehat{e}}}_2) =\begin{pmatrix} \cos \eta &{} 0 &{} \sin \eta \\ 0 &{} 1 &{} 0 \\ -\sin \eta &{} 0 &{} \cos \eta \end{pmatrix} \in SO(3), \quad&{\mathsf {e}}_2&= (0,1,0), \end{aligned}$$

and $\xi$ is the angle, about ${{\mathbf {t}}}$, measured from the principal normal ${{\mathbf {n}}}$ to ${{\mathbf {d}}}$ and $\eta$ is the angle, about ${{\mathbf {d}}}$, from the first tangent, ${{\mathbf {t}}}$, to the second, ${{\mathbf {t}}}_{a}$. From Eqs. (1) and (2) it follows that the rotation vectors of the various frames are related as3
4The relation Eq. (3) can be rewritten explicitly in component form:

5 $$\begin{aligned} \widetilde{\omega }_1&= \omega _1 - \xi ' , \end{aligned}$$

6 $$\begin{aligned} \widetilde{\omega }_2&= \omega _2 \cos \xi - \omega _3 \sin \xi , \end{aligned}$$

7 $$\begin{aligned} \widetilde{\omega }_3&= \omega _2 \sin \xi + \omega _3 \cos \xi . \end{aligned}$$

Before writing down the analogous component equations for Eq. (4) we express the angle $\eta$ as a function of the components of $\omega$ as follows. Differentiating the secondary curve ${{\mathbf {a}}}={{\mathbf {r}}}+\rho {{\mathbf {d}}}$ with respect to $s$ and using Eq. (1) yields

8 $$\begin{aligned} \sin \eta = -\frac{\rho \omega _1}{\sqrt{(\rho \omega _1)^2+(\rho \omega _3-1)^2}}, \quad \cos \eta = \frac{1-\rho \omega _3}{\sqrt{(\rho \omega _1)^2+(\rho \omega _3-1)^2}} , \end{aligned}$$

and hence

9 $$\begin{aligned} \omega _1 = \left( \omega _3 -\frac{1}{\rho }\right) \tan \eta . \end{aligned}$$

Equation (4) in component form can now be written as

10 $$\begin{aligned} \varOmega _1&= \omega _1 \cos \eta - \omega _3 \sin \eta = \frac{\omega _1}{\sqrt{(\rho \omega _1)^2+(\rho \omega _3-1)^2}},\end{aligned}$$

11 $$\begin{aligned} \varOmega _2&= \omega _2 + \eta ' = \omega _2 + \frac{\rho \omega '_1 (\rho \omega _3 -1) -\rho ^2 \omega _1 \omega '_3}{(\rho \omega _1)^2+(\rho \omega _3-1)^2} ,\end{aligned}$$

12 $$\begin{aligned} \varOmega _3&= \omega _1 \sin \eta +\omega _3 \cos \eta = \frac{\omega _3(1-\rho \omega _3)-\rho \omega _1^2}{\sqrt{ (\rho \omega _1)^2+(\rho \omega _3-1)^2}} . \end{aligned}$$

In summary, the Darboux frame $\{{{\mathbf {t}}},{{\mathbf {d}}},{{\mathbf {u}}}\}$ is always defined, the Frenet frame $\{{{\mathbf {t}}},{{\mathbf {n}}},{{\mathbf {b}}}\}$ and the angle $\xi$ are defined if the curvature $\varkappa$ does not vanish, and the secondary curve frame $\{{{\mathbf {t}}_{{\mathbf {a}}}},{{\mathbf {d}}},{{\mathbf {v}}}\}$ and the angle $\eta$ are defined if $(\rho \omega _1)^2+(\rho \omega _3-1)^2 > 0$. In this setup, the $\omega _i, \xi$ and $\eta$ are all $C^1$. Note that in this case the primary curve ${{\mathbf {r}}}(s)$ lies on a tubular surface of radius $|\rho |$ about the secondary curve ${{\mathbf {a}}}(s)$. In the following section we derive criteria for cylindrical curves by viewing such cylindrical curves as curves whose secondary curves are straight lines.

## Cylindrical curves

### Criterion for a curve to be cylindrical

#### **Proposition 1**

Let $C=\{{{\mathbf {r}}}(s), s\in [0,L]\}$ be a $C^3$-curve in ${{\mathbb R}}^3$.

(i) Let $\{ {{\mathbf {t}}}(s), {{\mathbf {d}}}(s), {{\mathbf {u}}}(s) \}$ be a moving frame along $C$ with ${{\mathbf {t}}}(s)$ the tangent and with Darboux vector  of class $C^1$ satisfying
  13 $$\begin{aligned} \omega _3&= \rho \left( \omega _1^2+\omega _3^2\right) ,\end{aligned}$$
  14 $$\begin{aligned} \omega '_3&= 2 \omega _1 \omega _2 \end{aligned}$$
  for some constant $\rho \ne 0$. Then the curve $C$ belongs to the surface of a circular cylinder of radius $|\rho |$. The vector ${{\mathbf {d}}}$ is normal to the surface and the components $\omega _i$ are, respectively, the geodesic torsion, geodesic curvature and normal curvature: $\omega _1=\tau _g, \omega _2=\varkappa _g, \omega _3=\varkappa _N$.
(ii) Conversely, if the curve $C$ lies on a circular cylinder of radius $\rho >0$, then its geodesic torsion $\tau _g$, geodesic curvature $\varkappa _g$ and normal curvature $\varkappa _N$ satisfy
  15 $$\begin{aligned} \varkappa _N&= \rho (\varkappa ^2_N + \tau ^2_g) ,\end{aligned}$$
  16 $$\begin{aligned} \varkappa '_N&= 2 \varkappa _g \tau _g . \end{aligned}$$

#### *Proof*

(i) We interpret ${{\mathbf {d}}}$ as a chord as in Sect. 2 defining a secondary curve ${{\mathbf {a}}}(s) = {{\mathbf {r}}}(s)+\rho {{\mathbf {d}}}(s)$ at constant separation $|\rho |$ to $C$. Consider first the case when $(\rho \omega _1)^2+(\rho \omega _3-1)^2 >0$. The tangent ${{\mathbf {t}}}_a$ to ${{\mathbf {a}}}$ is then defined and so is the moving frame $\{{{\mathbf {t}}}_a(s), {{\mathbf {d}}}(s), {{\mathbf {v}}}(s)\}$. This moving frame has Darboux vector  whose components are given by Eqs. (10)–(12). We see that after substitution of $\omega ^2_1$ from Eq. (13) the last component $\varOmega _3$ vanishes identically. By differentiating Eq. (13) and using the resulting equation together with Eq. (14) to eliminate the derivatives in the expression for $\varOmega _2$, we see that also $\varOmega _2=0$. Thus, the secondary curve has vanishing curvature, i.e., it is a straight line. This implies that the curve $C$ lies at constant separation $|\rho |$ from a straight line, i.e., it belongs to the surface of a cylinder of revolution with normal vector ${{\mathbf {d}}}(s)$. Now consider the case when $\omega _1(s)=0$ and $\omega _3(s)=1/\rho$ for some interval $s \in [s_1, s_2], s_2 > s_1$ (we only need to consider a single such interval). The secondary curve then shrinks to a point over this interval and the primary curve makes a circular arc of radius $|\rho |$, which clearly belongs to the surface of a circular cylinder. We need to show that this is the same cylinder as that containing the part of the curve for $0\le s<s_1$ (which is cylindrical by virtue of the first part of the proof). This is, however, ensured by the smoothness of the curve ${{\mathbf {r}}}\, (C^3)$ and the chord ${{\mathbf {d}}}\, (C^2)$: continuity of ${{\mathbf {d}}}$ implies that the axes of the two cylinders must intersect and continuity of the tangent ${{\mathbf {r}}}'$, and the fact that this tangent must be orthogonal to both axes at the joining point $s_1$, then implies that the two axes in fact coincide. One similarly shows that the curve remains on the same cylinder for $s > s_2$. The case of a single point where $\tau _g=0$ and $\varkappa _N=1/\rho$ is also treated similarly.
(ii) Let ${{\mathbf {d}}}(s)$ be the unit normal to the cylinder at $s$ along $C$. Then the moving frame $\{{{\mathbf {r}}}'(s), {{\mathbf {d}}}(s), {{\mathbf {u}}}(s)\}$ has Darboux vector $(\omega _1,\omega _2,\omega _3)=(\tau _g,\varkappa _g,\varkappa _N)$, where $\tau _g$ is the geodesic torsion, $\varkappa _g$ the geodesic curvature and $\varkappa _N$ the normal curvature [4]. Use the vector ${{\mathbf {d}}}(s)$, which is at least $C^2$ by smoothness of the cylindrical surface, as chord to construct the secondary curve ${{\mathbf {a}}}(s)={{\mathbf {r}}}(s)+\rho {{\mathbf {d}}}(s)$. Then this curve coincides with the cylinder axis. If $(\rho \tau _g)^2+(\rho \varkappa _N-1)^2 >0$ then the tangent ${{\mathbf {t}}}_a(s)$ and the secondary curve are defined and we can construct the moving frame $\{{{\mathbf {t}}}_a(s), {{\mathbf {d}}}(s), {{\mathbf {v}}}(s)\}$ with rotation vector  given by Eqs. (10)–(12). Vanishing curvature of the cylinder axis implies $\varOmega _2 \equiv 0$ and $\varOmega _3 \equiv 0$. The latter equation immediately yields Eq. (15), while from the first one we have
  $$\begin{aligned} 2 \varkappa _g \tau _g = -\frac{2\tau _g\left[ \rho \tau '_g (\rho \varkappa _N -1) -\rho ^2 \tau _g \varkappa '_N\right] }{(\rho \tau _g)^2+(\rho \varkappa _N-1)^2}. \end{aligned}$$
  Differentiating Eq. (15) and substituting $\tau _g$ and $\tau _g \tau '_g$ from Eq. (15) and its derivative into the right-hand side of the above expression we obtain $2 \varkappa _g \tau _g = \varkappa '_N$, which proves Eq. (16).
  If there exists an interval $[s_1,s_2], s_2>s_1$, such that $\tau _g(s)=0$ and $\varkappa _N(s) = 1/\rho$ for $s \in [s_1,s_2]$, then $\varkappa '_N(s)=0$ and Eqs. (15) and (16) are both satisfied in the interior of the interval, and by smoothness of ${{\mathbf {r}}}$ and ${{\mathbf {d}}}$ also over the entire interval $[0,L]$.
  Finally, consider isolated points where both $\tau _g=0$ and $\varkappa _N=1/\rho$. At such points the tangent to the curve is along the direction of maximum principal curvature (orthogonal to the axis of the cylinder) and therefore $\varkappa '_N=0$. Equations (15) and (16) are thus satisfied.

#### *Remark 1*

As noted in [4] (p. 193), the quantity $\varkappa '_N - 2 \varkappa _g \tau _g$, like $\varkappa _N$ and $\tau _g$ and unlike $\varkappa _g$, depends only on ${{\mathbf {t}}}(s)$. It is called the Laguerre function of direction. The curves on surfaces for which Eq. (16) holds are called Laguerre lines. These lines are the only curves that possess the following property: for every point of a Laguerre line the normal plane of the surface containing the tangent to the Laguerre line in this point, cuts the surface along another curve that has contact of the third order with its circle of curvature at this point. Indeed, for any direction of the tangent vector ${{\mathbf {t}}}$ different from the axis ${{\mathbf {t}}}_a$, the normal section of the cylinder is an ellipse and the point where this ellipse touches the cylindrical curve is a vertex of the ellipse. Thus, the curvature of the cross-sectional ellipse, which is $\varkappa _N$, is stationary in this point and the circle of curvature is superosculating to the ellipse, so any direction on the cylinder is along a Laguerre line. It is, therefore, not surprising that one of the conditions for a curve to be cylindrical is that the curve be a Laguerre line.

Proposition 1 gives a criterion in terms of geometric properties $(\tau _g, \varkappa _g$ and $\varkappa _N)$ of the curve as it lies in a surface. It may be desirable to have a criterion in terms of the intrinsic properties (i.e., curvature and torsion) of the curve. In the following two subsections we derive such criteria.

### Sufficient conditions for a curve to be cylindrical

#### **Proposition 2**

Let a $C^4$-curve $C=\{{{\mathbf {r}}}(s), s\in [0,L]\}$ in ${{\mathbb R}}^3$, parametrised by arclength, have curvature $\varkappa \ne 0$ and torsion $\tau$ satisfying the differential-algebraic system of two equations

17 $$\begin{aligned} \varkappa \cos \xi&= \rho [(\tau +\xi ')^2+\varkappa ^2 \cos ^2\xi ],\end{aligned}$$

18 $$\begin{aligned} \varkappa ' \cos \xi&= \varkappa (2\tau +3\xi ') \sin \xi , \end{aligned}$$

for some $C^2$-function $\xi (s)$ and for some constant $\rho \ne 0$. Then the curve $C$ belongs to the surface of a circular cylinder of radius $|\rho |$.

#### *Proof*

For nonvanishing curvature, the Frenet frame $\{ {{\mathbf {t}}}(s), {{\mathbf {n}}}(s), {{\mathbf {b}}}(s) \}$ is well defined. Define another moving frame $\{ {{\mathbf {t}}}(s), {{\mathbf {d}}}(s), {{\mathbf {u}}}(s) \}$ that is obtained from the Frenet frame by rotation about the tangent through the angle $\xi (s)$ as defined in Sect. 2. Note that since ${{\mathbf {n}}}(s)$ and $\xi (s)$ are both $C^2$, the vector ${{\mathbf {d}}}(s)$ is also $C^2$, as required. Then by Eqs. (5)–(7) this frame has Darboux vector $(\omega _1,\omega _2,\omega _3)=(\tau +\xi ',\varkappa \sin \xi ,\varkappa \cos \xi )$. Substitution into Eqs. (17)–(18) gives Eqs. (13)–(14) and the claim follows from Proposition 1(i).

#### *Remark 2*

We can eliminate $\xi '$ from Eqs. (17)–(18) and obtain an equation of the form $F(\varkappa ,\tau ,\varkappa ',\xi )=0$. From this equation we can find (at least numerically) $\tan \xi$ as a root of an eighth-degree polynomial with coefficients that are functions of $\varkappa , \tau$ and $\varkappa '$. Formally differentiating this solution $\tan \xi$ and using one of Eqs. (17)–(18) we can then arrive at an equation of the form $G(\varkappa ,\tau ,\varkappa ',\tau ',\varkappa '')=0$, with dependence on derivatives of $\varkappa$ and $\tau$ up to the same order as in [2].

In applications such as computer vision it may be useful to have a criterion for cylindrical curves not involving the radius $\rho$, which may be a priori unknown. $\rho$ may be eliminated from Eqs. (17)–(18), at the cost of higher derivatives, by solving the first equation with respect to $\rho$ and differentiating the resulting expression with respect to $s$.

The characterisation of cylindrical curves in Proposition 2 involves a differential equation. Explicit conditions for $\varkappa$ and $\tau$ can be derived by using the angle $\eta$ (or its lift) instead of the angle $\xi$, as follows.

#### **Proposition 3**

Let a $C^4$-curve $C=\{{{\mathbf {r}}}(s), s\in [0,L]\}$ in ${{\mathbb R}}^3$, parametrised by arclength, have curvature

19 $$\begin{aligned} \varkappa =\sqrt{\theta '^2+\frac{\sin ^4\theta }{\rho ^2}} > 0 \end{aligned}$$

and torsion

20 $$\begin{aligned} \tau =\frac{\sin \theta [\rho ^2(\theta ''\sin \theta -3\theta '^2\cos \theta )-\sin ^4\theta \cos \theta ]}{\rho (\rho ^2\theta '^2+\sin ^4\theta )}, \end{aligned}$$

for some $C^3$-function $\theta (s)$ and some constant $\rho \ne 0$. Then the curve $C$ belongs to the surface of a circular cylinder of radius $|\rho |$ and $\theta$ is the angle between the tangent to the curve and the axis of the cylinder.

#### *Proof*

The curvature $\varkappa$ of $C$ does not vanish, hence the Frenet frame $\{{{\mathbf {t}}},{{\mathbf {n}}},{{\mathbf {b}}}\}$ and the torsion $\tau$ are well defined. We construct a secondary curve as follows. First we define a moving frame $\{ {{\mathbf {t}}}(s), {{\mathbf {d}}}(s), {{\mathbf {u}}}(s) \}$ by rotating the Frenet frame through an angle

$$\begin{aligned} \xi := \arctan \left( -\frac{\theta '}{\varkappa }, \frac{\sin ^2\theta }{\rho \varkappa }\right) \end{aligned}$$

about the tangent ${{\mathbf {t}}}$. Note that since ${{\mathbf {n}}}(s)$ and $\xi (s)$ are both $C^2$, the vector ${{\mathbf {d}}}(s)$ is also $C^2$, as required. We compute the components of the rotation vector of the frame $\{{{\mathbf {t}}},{{\mathbf {d}}},{{\mathbf {u}}}\}$ as

21 $$\begin{aligned} \omega _1&= \tau + \xi ' ,\end{aligned}$$

22 $$\begin{aligned} \omega _2&= \varkappa \sin \xi = -\theta ',\end{aligned}$$

23 $$\begin{aligned} \omega _3&= \varkappa \cos \xi = \frac{1}{\rho } \sin ^2\theta \end{aligned}$$

[the inverse of Eqs. (5)–(7)] and easily verify that Eqs. (13)–(14) are satisfied. The claim then follows from Proposition 1(i).

#### *Remark 3*

Unlike spherical curves, cylindrical curves can have vanishing curvature. A straight line belongs to a continuum of cylinders aligned with the line. The presence of a straight interval between curvilinear pieces can make these curvilinear pieces belong to different cylinders. The characterisation of cylindrical curves in terms of curvature and torsion stops working in this situation. That is why the requirement of nonvanishing curvature appears in the statement of Propositions 2 and 3.

### Necessary conditions for a curve to be cylindrical

#### **Proposition 4**

Let a $C^3$-curve $C=\{{{\mathbf {r}}}(s), s\in [0,L]\}$ in ${{\mathbb R}}^3$, parametrised by arclength, belong to the surface of a circular cylinder of radius $\rho >0$. Then there exists a $C^1$-function $\xi (s)$ such that the curvature $\varkappa$ and torsion $\tau$ satisfy the differential-algebraic system of two equations

24 $$\begin{aligned} \varkappa \cos \xi&= \rho [(\tau +\xi ')^2+\varkappa ^2 \cos ^2\xi ] ,\end{aligned}$$

25 $$\begin{aligned} \varkappa ' \cos \xi&= \varkappa (2\tau +3\xi ') \sin \xi \end{aligned}$$

in all points where $\varkappa \ne 0$.

#### *Proof*

If $\varkappa \ne 0$ then the Frenet frame is defined. We choose for ${{\mathbf {d}}}(s)$ the vector normal to the cylinder at a point $s$ along $C$. The secondary curve ${{\mathbf {a}}}={{\mathbf {r}}}+\rho {{\mathbf {d}}}$ then coincides with the axis of the cylinder and the rotation vector of the moving frame $\{{{\mathbf {r}}}',{{\mathbf {d}}},{{\mathbf {u}}}\}$ along the primary curve has components $(\omega _1,\omega _2,\omega _3)=(\tau _g,\varkappa _g,\varkappa _N)$. We first consider intervals where $(\rho \omega _1)^2+(\rho \omega _3-1)^2 \ne 0$, so that the rotation vector of the secondary curve $(\varOmega _1,\varOmega _2,\varOmega _3)$ and the angle $\eta \in (-\pi /2, \pi /2)$ are well-defined [see Eqs. (8)–(12)]. Since the secondary curve is a straight line we have $\varOmega _2=0=\varOmega _3$. Equations (9)–(12) then imply

26 $$\begin{aligned} \tau _g&=-\frac{1}{\rho } \sin \eta \cos \eta ,\end{aligned}$$

27 $$\begin{aligned} \varkappa _g&= - \eta ' ,\end{aligned}$$

28 $$\begin{aligned} \varkappa _N&= \frac{1}{\rho }\sin ^2\eta . \end{aligned}$$

To write these conditions in terms of curvature $\varkappa$ and torsion $\tau$ of the curve we use Eqs. (5)–(7) to get

29 $$\begin{aligned} \tau +\xi '&= -\frac{1}{\rho }\sin \eta \cos \eta ,\end{aligned}$$

30 $$\begin{aligned} \varkappa \sin \xi&= -\eta ',\end{aligned}$$

31 $$\begin{aligned} \varkappa \cos \xi&= \frac{1}{\rho }\sin ^2\eta , \end{aligned}$$

where $\xi (s)$ is the angle between the principal normal ${{\mathbf {n}}}(s)$ and ${{\mathbf {d}}}(s)$ along $C$. According to Eq. (5), for a $C^3$-curve, $\xi (s)$ is a $C^1$-function. By eliminating $\eta$ from these equations we obtain Eqs. (24)–(25).

Now consider the case that, for some interval $[s_1, s_2], 0 \le s_1 < s_2 \le L$, both $\tau _g=0$ and $\varkappa _N=1/\rho$. Then, over this interval, the curve is a circular arc with curvature $\varkappa = 1/\rho =\mathop {\mathrm{const}}\nolimits$, torsion $\tau =0$ and angle $\xi$ identically equal to zero. Equations (24)–(25) are thus satisfied in the interior of the interval and by smoothness also at $s=s_1$ and $s=s_2$. By the same smoothness argument the equations are also satisfied if $\tau _g=0$ and $\varkappa _N=1/\rho$ at an isolated point $s=s_1$.

#### **Proposition 5**

Let a $C^3$-curve $C=\{{{\mathbf {r}}}(s), s\in [0,L]\}$ in ${{\mathbb R}}^3$, parametrised by arclength, belong to the surface of a circular cylinder of radius $\rho >0$. Then there exists a $C^2$-function $\theta (s)$ such that the curvature equals

32 $$\begin{aligned} \varkappa =\sqrt{\theta '^2+\frac{\sin ^4\theta }{\rho ^2}} \end{aligned}$$

and, in points where $\varkappa \ne 0$, the torsion equals

33 $$\begin{aligned} \tau =\frac{\sin \theta [\rho ^2(\theta ''\sin \theta -3\theta '^2\cos \theta )- \sin ^4\theta \cos \theta ]}{\rho (\rho ^2\theta '^2+\sin ^4\theta )} . \end{aligned}$$

$\theta$ is the angle between the tangent to the curve and the axis of the cylinder.

#### *Proof*

Following the proof of Proposition 4 we choose the normal to the cylinder as our ${{\mathbf {d}}}(s)$ and construct a moving frame and secondary curve that coincides with the axis of the cylinder. For nonvanishing curvature and on intervals where $(\rho \omega _1)^2+(\rho \omega _3-1)^2 \ne 0$ we again arrive at Eqs. (29)–(31), but now eliminate $\xi$ to obtain

34 $$\begin{aligned} \rho ^2(\varkappa ^2-\eta '^2)&= \sin ^4\eta ,\end{aligned}$$

35 $$\begin{aligned} \varkappa '\sin ^2\eta&= \varkappa \eta ' (\rho \tau + 3 \sin \eta \cos \eta ) . \end{aligned}$$

Solving Eq. (34) for the curvature gives Eq. (32) for $\theta = \eta + \pi n, n \in {{\mathbb Z}}$, where $n$ is chosen to make $\theta$ continuous. Differentiating Eq. (32) to obtain $\varkappa '$, substituting the result into Eq. (35) and solving for the torsion gives Eq. (33). For a $C^3$ curve, $\eta$ (and hence $\theta$) is generally $C^1$. However, Eq. (34) [or Eq. (33)] shows that for the present case of a cylindrical curve, $\theta$ is in fact $C^2$.

If the curvature vanishes on an interval then the tangent ${{\mathbf {t(s)}}}$ must be aligned with the direction of least principal curvature on the cylindrical surface, i.e., parallel to the axis of the cylinder and we have $\eta =0$, and also $\eta '=0$, and hence $\theta '=0$, in the interior of the interval, so Eq. (32) holds. At the end points of the interval we can use smoothness to take limits and thus show that Eq. (32) holds too. Similarly, in isolated points of zero curvature, the angle $\eta$ (and hence $\theta$) is, strictly speaking, not defined, but we can take limits and set $\theta =0=\theta '$ in such points to obtain a $C^2$-function $\theta (s)$.

Intervals or points where both $\tau _g=0$ and $\varkappa _N=1/\rho$ are dealt with in the same way as in the proof of Proposition 4.

#### *Remark 4*

It is perhaps unexpected that in the sufficiency propositions of Sect. 3.2 the curve ${{\mathbf {r}}}(s)$ is required to be $C^4$, while in the necessity propositions of Sect. 3.3 it is only required to be $C^3$. The reason is that in the former we have no (tubular) surface until we have constructed a chord vector ${{\mathbf {d}}}$, for which we use the Frenet frame of the curve, while in the latter we are given a smooth surface (a cylinder) and use this to construct a ${{\mathbf {d}}}$. This ${{\mathbf {d}}}(s)$ must be $C^2$ for the curvatures $\varOmega _i$ of the secondary curve to be defined, so the principal normal ${{\mathbf {n}}}(s)$ of the Frenet frame must also be $C^2$ and hence ${{\mathbf {r}}}(s)$ must be $C^4$, while on a smooth cylinder a $C^3$-curve is sufficient to guarantee the required smoothness of ${{\mathbf {d}}}(s)$.

#### *Remark 5*

Equations (34)–(35) are essentially those in [3] (after elimination of the second derivative).

## A sufficient condition for a cylindrical curve to be closed

In [1] a necessary and sufficient condition was given for a planar curve of periodic curvature to be closed. Since a cylinder is a developable surface this result immediately gives a sufficient condition for a cylindrical curve to be closed provided we replace (signed) curvature by geodesic curvature:

### **Proposition 6**

Let $C=\{{{\mathbf {r}}}(s), s\in [0,L]\}$ be a $C^3$-curve in ${{\mathbb R}}^3$ lying on the surface of a (not necessarily circular) cylinder and let the geodesic curvature $\varkappa _g$ have (minimum) period $P$. Then the curve $C$ closes up in $[0,nP], n\in {{\mathbb N}}, n>1$, if there exists an integer $m \in {{\mathbb Z}}$ such that

36 $$\begin{aligned} \frac{1}{2\pi } \int \nolimits _0^P \varkappa _g \ \text{ d }s =\frac{m}{n} \in {{\mathbb Q}}\backslash {{\mathbb Z}} \ . \end{aligned}$$

Note that we cannot claim Eq. (36) to be a necessary condition because of the possibility of closed curves that wrap around the cylinder over which we have no control [indeed, a circular curve around the cylinder has $\varkappa _g=0$ and so Eq. (36) is not satisfied]. However, Eq. (36) is a necessary condition if we restrict ourselves to closed curves that are contractible (within the cylindrical surface) to a point.

## Example

**Fig. 1:**
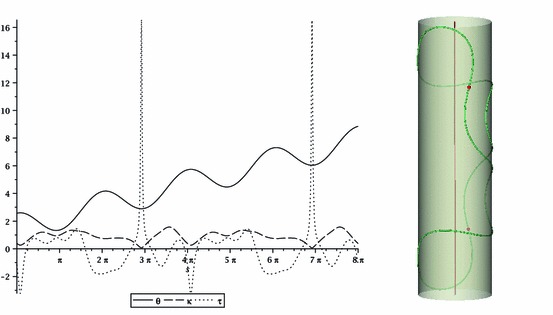
*Left* function $\theta (s)=\pi /2+\cos {s}+s/4, s \in [0, 8\pi ]$ and the curvature $\kappa$ and torsion $\tau$ as given by Eqs. (32) and (33). *Right* corresponding curve on the surface of a cylinder $(\rho =1)$. *Red beads mark points* of minimum curvature, zero geodesic curvature and maximum torsion (colour figure online)

We illustrate here how the conditions in the previous two sections work in a simple example. We take $\rho =1$ and choose $\theta (s)=\pi /2+\cos {s}+s/4$. Then, from Eqs. (26)–(28), we have $\tau _g =-\sin \theta \cos \theta , \varkappa _g =-\theta '=\sin s -\frac{1}{4}$ and $\varkappa _N = \sin ^2\theta$. We also compute the curvature and torsion by applying Eqs. (32), (33) (see Fig. 1, left). From this we compute the curve by integrating the Frenet–Serret equations together with ${{\mathbf {r}}}'(s)={{\mathbf {t}}}(s)$. The resulting contractible closed curve lies on the surface of a cylinder of unit radius (see Fig. 1, right). Note that the curvature never vanishes and the torsion reaches a sharply peaked maximum when the curvature is at a minimum and $\varkappa _g=0$.

Proposition (6) gives a condition on integers $m$ and $n$ for a cylindrical curve with $\theta (s)=\frac{\pi }{2}+\cos {s}+\frac{m}{n} s$ to be closed. The geodesic curvature of this curve is $\varkappa _g = -\theta ' = \sin (s)-\frac{m}{n}$, which has minimum period $P=2 \pi$. In our particular example $m=1, n=4$, so the curve closes up in $[0, 8\pi ]$.
